# Meta‐analysis and meta‐regression of transcriptomic responses to water stress in Arabidopsis

**DOI:** 10.1111/tpj.13124

**Published:** 2016-02-12

**Authors:** Joshua S. Rest, Olivia Wilkins, Wei Yuan, Michael D. Purugganan, Jessica Gurevitch

**Affiliations:** ^1^ Department of Ecology and Evolution, 650 Life SciencesStony Brook UniversityStony BrookNY11794–5245USA; ^2^ Center for Genomics and Systems BiologyNew York University12 Waverly PlaceNew YorkNY10003USA

**Keywords:** *Arabidopsis thaliana*, drought response, water stress, meta‐analysis, research synthesis, gene expression microarray, transcriptomics

## Abstract

The large amounts of transcriptome data available for *Arabidopsis thaliana* make a compelling case for the need to generalize results across studies and extract the most robust and meaningful information possible from them. The results of various studies seeking to identify water stress‐responsive genes only partially overlap. The aim of this work was to combine transcriptomic studies in a systematic way that identifies commonalities in response, taking into account variation among studies due to batch effects as well as sampling variation, while also identifying the effect of study‐specific variables, such as the method of applying water stress, and the part of the plant the mRNA was extracted from. We used meta‐analysis, the quantitative synthesis of independent research results, to summarize expression responses to water stress across studies, and meta‐regression to model the contribution of covariates that may affect gene expression. We found that some genes with small but consistent differential responses become evident only when results are synthesized across experiments, and are missed in individual studies. We also identified genes with expression responses that are attributable to use of different plant parts and alternative methods for inducing water stress. Our results indicate that meta‐analysis and meta‐regression provide a powerful approach for identifying a robust gene set that is less sensitive to idiosyncratic results and for quantifying study characteristics that result in contrasting gene expression responses across studies. Combining meta‐analysis with individual analyses may contribute to a richer understanding of the biology of water stress responses, and may prove valuable in other gene expression studies.

## Significance Statement

Transcriptome studies are sometimes difficult to compare due to different experimental conditions. Here we show that that meta‐analysis and meta‐regression are useful for identifying robust gene sets and can quantify study characteristics that result in contrasting gene expression responses across studies. Combining meta‐analysis with individual analyses thus contribute to a richer understanding of the biology of water stress responses and may prove valuable in other gene expression studies.

## Introduction

Differentially expressed genes (DEGs) are important indicators of plant physiological responses to different environments. Transcriptome‐wide studies enable comprehensive enumeration of DEGs and their magnitude of change between paired treatments. While much of the current focus is on RNA‐seq data, a great amount of data obtained using microarray methodology exists, addressing many important types of plant response. The large amounts of transcriptome data available for the model plant species *Arabidopsis thaliana* make a compelling case for the need to generalize results across studies to extract the most robust and meaningful information possible from this large body of information. Since 2003, more than 800 data sets generated using Affymetrix ATH1 whole‐genome microarrays have been deposited in the Gene Expression Omnibus. These data sets have examined gene expression in response to various environmental factors, including insect herbivory, heat stress, water stress and pathogens, as well as gain‐of‐function and loss‐of‐function mutations and developmental processes. Gene expression results have rarely been quantitatively synthesized across studies for Arabidopsis or for any other plant species (Postnikova and Nemchinov, 2012; Rodrigo *et al*., 2012; but see Shaik and Ramakrishna, 2013). Plant microarray studies are sometimes compared by listing DEGs identified by different studies and counting the number of genes identified in common; the results of these comparisons are often presented using Venn diagrams to show overlaps in DEGs discovered by various studies. Other studies have analyzed all arrays simultaneously using *t* tests or anova, or have transformed expression values into ranks (Yu *et al*., 2011; Bhargava *et al*., 2013; Lai and Ge, 2014). These approaches have in common a key limitation: they are not able to account for the variance in responses across biological replicates in an accurate and unbiased manner. In addition, they do not account for difference in the precision with which responses are measured across studies (i.e. the inverse of the study sampling variance; studies with two biological replicates are counted as heavily as those with 20 biological replicates, although the confidence intervals of the latter study would be much smaller). Sampling variance is a statistical concept that estimates the amount of random variation in outcomes that would be expected if one repeated the same experiment in the same way multiple times. It is estimated from the number of biological replicates and the variation among them in a given study, and has a larger value when the number of replicates is small. Additional sources of variation in gene expression among studies include batch effects due to unknown factors (Kang *et al*., 2011), as well as known biological and methodological differences that have been measured and reported, such as differences in plant treatment and study methodology. Modern methods for meta‐analysis and meta‐regression can account for these different sources of variance statistically, thereby reducing bias due to study‐specific factors and small sample sizes. By contrast, some of the conventional approaches used to synthesize study results suffer from statistical biases that may even become more pronounced with increased amounts of data (Gurevitch and Hedges, 1999; Koricheva and Gurevitch, 2014). Due to these and other limitations, results may seem inconsistent, contradictory or heterogeneous across studies, even when they actually are consistent.

The statistically rigorous methods of meta‐analysis and meta‐regression address the limitations of older approaches to cross‐study comparisons, and are used by scientists in many fields who are interested in quantitative research synthesis. In a formal meta‐analysis, the outcomes of various studies addressing the same question in comparable ways are expressed on the same scale, using a metric known as ‘effect size’. For example, the effect size may be a differential response to an experimental treatment, expressed as the ratio of the mean response to an experimental treatment to the mean response in a control. The outcomes of each study, expressed in terms of the effect sizes, are then combined. However, rather than taking the simple mean, the studies are combined by weighting more precise estimates of the studies’ effect sizes more heavily than those of smaller and more variable studies. In doing so, variation both within and among studies is accounted for. Because each study is weighted inversely to its variance, this is called inverse‐variance weighted meta‐analysis. Statistical models may then be used to account for true random variation among study outcomes, and for hypothesized sources of variation using meta‐regression (described below).

Here, we use inverse‐variance weighted meta‐analysis to synthesize the results of differential gene expression studies on the responses of Arabidopsis to water stress, based on extending conventional meta‐analysis approaches to transcriptomic data. The effect sizes are based on differential gene expression. While differential gene expression is typically expressed as log_2_ fold change, according to the convention in meta‐analysis (Hedges *et al*., 1999), we use the closely related effect size metric called the log expression ratio (ln*R*). This is the natural log of the ratio of the mean expression level of a gene (i.e. probe set) in an experimental group to the mean expression level of the gene in a control group.

Meta‐analysis approaches have recently begun to be applied to gene expression studies in medical and other research fields, and have been shown to reduce inconsistencies, account for sampling variation, and detect candidate genes that are differentially expressed consistently across studies (Stevens and Doerge, 2005; Hong and Breitling, 2008; Kang *et al*., 2011; Tseng *et al*., 2012). Instead of weighting based on variance, earlier approaches to synthesize gene expression studies included rank‐based tests and combining *P* values (Hong and Breitling, 2008), and several R packages are based on these methods (e.g. Hong *et al*., 2006). While there are few such studies in plants, one that we are aware of combined differential gene expression results to drought and bacterial stress in Arabidopsis and rice using both a rank‐based non‐parametric as well as a co‐expression approach (Shaik and Ramakrishna, 2013). A study by Zaag *et al*. (2015) used a model‐based clustering approach to find and visualize co‐expressed genes involved in the response of Arabidopsis to stress; this approach was applied to each of 18 stress categories. However, these approaches are limited because they do not account for differential variance among experiments, nor do they model methodological and biological covariates within or across stress categories. While each of these approaches has strengths, inverse‐variance weighted meta‐analysis has been shown to be an especially robust, comprehensive and consistent method of meta‐analysis for gene expression data (Ramasamy *et al*., 2008). Hereafter, when we discuss meta‐analysis, we are referring specifically to inverse‐variance weighted meta‐analysis, as used in our study. One of the first papers to introduce this approach to gene expression was a synthesis of cancer profiling studies (Choi *et al*., 2003). To our knowledge, this approach has not previously been applied to gene expression studies in plants. As discussed above, the advantage of using this approach is that, in addition to determining the overall response for each gene across studies, we can ensure that the effect is consistent across studies beyond random sampling variation, because it distinguishes sampling variation within studies (i.e. chance differences in the outcome that would occur if the same study was repeated in exactly the same way many times) from true heterogeneity among studies.

An extension of meta‐analysis, called meta‐regression, may be used to model the effects of hypothesized factors that may account for variation in responses across studies (Nakagawa and Santos, 2012; Mengersen and Schmid, 2013). In a meta‐regression, variation due to study characteristics (called moderators or covariates) may be modeled statistically, thereby accounting for heterogeneity in study outcomes (in addition to the random and sampling variation, described above, including batch effects, described below). Such covariates may be categorical or continuous factors that are biologically meaningful, such as the strain used or the temperature at which an experiment was performed, or methodological, such as the duration of the experiments. Meta‐regression may identify statistically significant covariates (i.e. factors that may explain meaningful heterogeneity among studies) and estimate their magnitude and sign (i.e. negative or positive effects on the study outcomes). Meta‐regression has been applied to date largely in medical research synthesis, and, to our knowledge, has not yet been applied to gene expression data. It offers a statistically powerful method for identifying factors that may alter responses. Meta‐regression was first introduced to research synthesis in medicine in the early to mid‐1990s (Schmid *et al*., 1994; Holme, 1996) with the primary goal of adjusting estimates of main effects, but its use has more recently been expanded, particularly in fields where understanding the contributions of covariates is of primary interest scientifically. In addition to using meta‐analysis to synthesize transcriptomic studies on water stress in Arabidopsis, we performed meta‐regression on transcriptomic data to draw inferences about specific responses attributable to biological and methodological characteristics of various studies.

The approaches described here may be adapted for any differential gene expression data, including both microarray and RNA‐seq data. The large amount of available microarray data, and the statistical benefits offered by meta‐analysis and meta‐regression, create a compelling argument for applying meta‐analysis to synthesize plant microarray studies. Importantly, meta‐analysis has the potential to identify not only DEGs with large differential responses (when these responses are consistent across studies), but also to discover DEGs with relatively small responses that are highly consistent within and across studies (Choi *et al*., 2003). On the other hand, genes with large but variable and inconsistent differential responses may be rejected as DEGs. The development of meta‐analysis and meta‐regression methodology holds promise for de‐convoluting effects specific to individual transcriptomic studies from more general, biologically important phenomena. Identifying sources of heterogeneity among studies may be highly informative in the biological interpretation of gene expression responses.

Compared to typical meta‐analysis data, microarray data have a very unusual and distinctive statistical structure, with very few replicates within studies and very large numbers of outcomes (gene expression values). All genome‐wide expression studies present the need to adjust for such large numbers of statistical tests on the same data set, requiring methods to account for false discoveries when performing thousands of statistical tests on the thousands of expressed genes (Ramasamy *et al*., 2008; Tseng *et al*., 2012). In single‐study genomic analyses, this has been addressed by using false discovery rate (FDR) analyses to adjust the results of multiple *t* tests and ANOVAs. Meta‐analyses of these data face the same issues, exacerbated by having multiple studies. Meta‐analysis outcomes must therefore also be corrected using the FDR to correct for multiple‐testing errors.

Statistical analysis of microarray studies also requires substantial technical quality control and normalization among replicates prior to research synthesis. After quality control and normalization, effect sizes are calculated, and statistical models are used to synthesize and analyze the responses across studies. As in any meta‐analysis on data in any field, various aspects of the studies will result in differences in the outcomes (i.e. the effect sizes): both those that are of scientific interest and those that must be accounted for but are not in themselves of interest. Early meta‐analyses used fixed‐effects statistical models, which assume that the outcomes of different studies differ only from one another in terms of sampling error, i.e. if all of the studies were ‘sufficiently’ large in size, the outcomes would be identical (e.g. Mengersen *et al*., 2013). However, it was recognized that many real (but unmeasured and uncharacterized) differences that result in differences in the outcomes may also exist among studies. To account for this, random‐effects models were developed (Mengersen *et al*., 2013). In transcriptomic data, for example, one such cause of variation among outcomes is batch effects. Batch effects are expression differences between samples as a result of unintentional experimental or technical heterogeneity in sample processing (e.g. between days, laboratories or platforms), and are known to affect both microarray approaches (Lander, 1999) and next‐generation sequencing approaches (Auer and Doerge, 2010). In some cases, the sources of such heterogeneity are in themselves of interest, and, if the data are available, they may be formally tested (as covariates in a meta‐regression model). However, in many cases, these sources of heterogeneity are not in themselves of particular interest, nor are the data available to test them formally; consequently they may be modeled and accounted for in the random error component of a meta‐analysis or meta‐regression model. We therefore treat the unknown sources of variation responsible for batch effects as a component of the random error variation among studies in our analyses, although other statistical approaches have also been suggested to account for batch effects (e.g. Johnson *et al*., 2007; Chen *et al*., 2011).

One advantage of the distinctive structure of microarray data is that, unlike other kinds of data (i.e. different medical studies performed on different groups of patients, measured using different instruments), the measurement methodology (i.e. the Affymetrix platform) is uniform, greatly reducing variation among studies. When based on a single platform (such as Affymetrix), the data have essentially identical measures (i.e. identical probes) across studies. This is very different from typical meta‐analysis data where measurements of outcome may differ considerably among studies.

The goal of the present study was to synthesize the results of differential expression microarray experiments in which Arabidopsis plants (Col–0 accession) were exposed to water stress. We used meta‐analysis and meta‐regression to identify patterns of gene expression across studies, and to determine which genes were expressed differently in response to several hypothesized biological and methodological covariates. In particular, we were interested in differences in responses in roots versus shoots (leaves and rosettes) and in response to different methods of inducing water stress. A separate meta‐analysis was performed for each gene, and the results were adjusted for the FDR. We then compare the results of the meta‐analysis to results obtained using the more standard approach of applying tests for differential expression to each individual study.

## Results

### Meta‐analysis reveals consistent responses to water stress across studies

**Figure 1:**
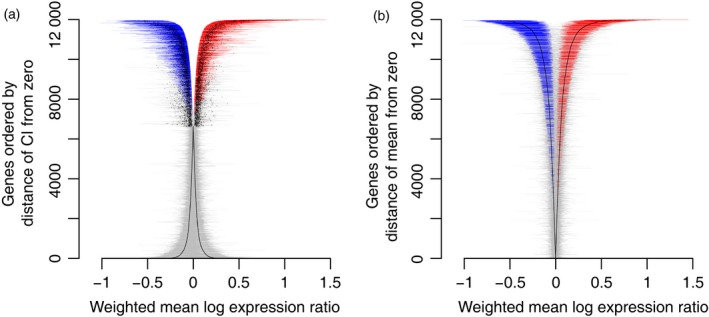
Meta‐analysis of the weighted mean of log expression ratios (i.e. weighted mean effect size, $\hat{\mu }$) of gene expression responses across water stress experiments, sorted by distance of the 95% confidence interval from zero (a), or distance of the weighted mean expression ratio from zero (b). Genes with confidence intervals that overlap zero in (a) are also sorted by the distance of the mean from zero. Each gene is indicated by a dot ($\hat{\mu }$) and a horizontal line (95% CI). Genes with significantly (FDR < 0.05) positive values of $\hat{\mu }$ (up‐regulated) are colored red; genes with significantly negative values of $\hat{\mu }$ (down‐regulated) are colored blue, and genes with values of $\hat{\mu }$ that are not significantly different from zero are colored gray. For genes with 95% CIs that overlap zero in (a), and for all genes in (b), the dots for $\hat{\mu }$ tend to merge into lines.

We performed a meta‐analysis of ten studies of gene expression responses to water stress in Arabidopsis (see Table 1) for each of 11 984 probe sets (hereafter referred to as genes) using a random‐effects model, and then corrected for multiple testing. Our meta‐analysis of these ten studies identified 4015 genes showing significant differential expression in response to water limitation compared to control conditions in *Arabidopsis thaliana* (FDR < 0.05; Figure S1a and Table S1). We constructed two novel versions of conventional meta‐analysis forest plots (e.g. Borenstein *et al*., 2011) designed to illustrate the responses for all of the DEG results (Figure 1a,b). In these figures, the results of the meta‐analysis for the responses of each of the 11 984 genes is represented by a single line with a symbol indicating the weighted mean effect size across studies ($\hat{\mu }$; i.e. the mean response for that gene across studies) and the 95% confidence interval (CI) around that mean. The two figures highlight different aspects of the overall response as well as specific responses. Figure 1(a) was designed to show the magnitude of the response for each gene as well as how far the CI for each of the 11 984 genes was from zero. A large value for $\hat{\mu }$ indicates a large mean response for the gene. If the CI overlaps zero, this indicates that there is no significant response for that gene, but if the CI is far from zero, it indicates confidence that the gene response was significant. To construct Figure 1(a), we sorted genes by the magnitude of the size of the gap between the edge of the CI for each gene and zero, for those responses that did not overlap zero. Those genes with CIs that overlapped zero were sorted below the first group of genes, by the magnitude of $\hat{\mu }$ (Figure 1a). In Figure 1(b), we re‐visualized the same results by sorting all gene responses by the magnitude of $\hat{\mu }$ for each of the genes (without consideration of the magnitude or value of the CI). The results for genes with values of $\hat{\mu }$ that are significantly different from zero in Figure 1(a,b) are colored either red (up‐regulated) or blue (down‐regulated); values of $\hat{\mu }$ that are not significantly different from zero are colored gray. An examination of Figure 1(a,b) shows that the ability to detect significant effects depends both on the magnitude of the CI (reflecting the repeatability of the findings across experiments) as well as the magnitude of $\hat{\mu }$. Thus, genes with large differential responses may not be significantly different from zero, if their CIs are very large, while genes with small but highly consistent effects across studies may be discovered because their tight CIs identify responses that are significantly different from zero (Choi *et al*., 2003). These genes are not as easily recognized by most conventional methods (e.g. *t* tests or anovas of individual studies). We compare the likely number of such discoveries of small but consistent differential responses below by comparing the meta‐analysis results to results from individual studies, and discuss some specific examples of genes with this outcome.

**Table 1:** Data sets used in meta‐analysis and number of differentially expressed genes (DEGs), according to individual *t* test contrasts and the intersection of each contrast and the meta‐analysis

Contrast	GEO accession	Plant part	Treatment	Number of arrays	DEGs by *t* test contrasts	Intersection of DEGs by *t *tests and meta‐analysis
A	GSE40061	Root	Water withholding	6	7988	2660
B	GSE40061	Leaf	Water withholding	6	3339	1436
C	GSE36789	Root	Mannitol	6	3627	1301
D	GSE36789	Leaf	Mannitol	6	2159	1139
E	GSE35258	Seedling	Polyethylene glycol	6	4257	2133
F	GSE19700	Rosette	Water withholding	6	0	0
G	GSE15577	Rosette	Water withholding	4	5068	2705
H	GSE10670	Leaf	Water withholding	6	3518	1912
I	GSE10643	Rosette	Water withholding	4	0	0
J	GSE6583	Rosette	Deracination	6	5080	2445

### Meta‐regression reveals plant part‐ and method‐specific responses

**Figure 2:**
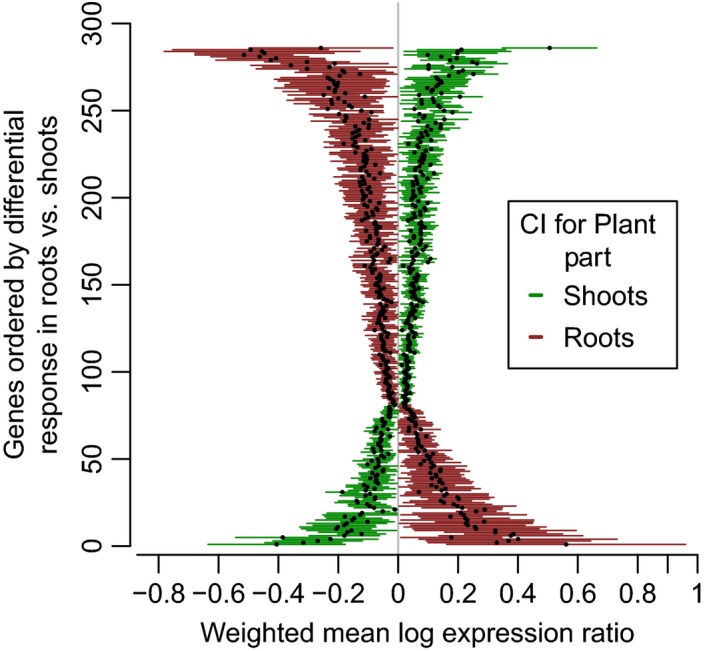
Differences in gene expression responses between shoots and roots identified using meta‐regression. Weighted means of log expression ratios ($\hat{\mu }$) for each gene are summarized in roots (red) and shoots (green), which are horizontally adjacent to each other. The value of $\hat{\mu }$ for each gene is indicated by a dot, with a horizontal line encompassing the 95% confidence interval (CI). The top half of the plot includes 167 genes that are up‐regulated in shoots and down‐regulated in roots, while the bottom half of the plot shows 76 genes that are down‐regulated in shoots and up‐regulated in roots.

We examined plant part‐specific responses to water stress by meta‐regression, using a random‐effects model with plant part included as a covariate in the model for each of the 11 984 genes. Specifically, after correction for multiple testing, we identified genes that had a significant non‐zero response to water stress in shoots or in roots, to determine genes for which these effects in shoots and roots are significantly different from each other. Using a random‐effects meta‐regression model with plant part as a covariate, we found 286 genes that are up‐regulated in one plant part and down‐regulated in another (FDR < 0.05; Figure 2, Figure S1b and Table S2). We also identified genes that are up‐regulated to different extents in response to water stress in shoots and roots, including three genes that are significantly more up‐regulated in shoots than in roots, and 22 genes that are significantly more up‐regulated in roots than in shoots. We also identified genes that are down‐regulated to different extents in response to water stress in shoots and roots, including four genes that are significantly more down‐regulated in shoots than in roots, and 87 genes that are significantly more down‐regulated in roots than in shoots.

Using the same approach, we performed a meta‐regression to examine the influence of the particular experimental method used to impose water stress (deracination, mannitol or water withholding) as a covariate in a random‐effects meta‐regression model. The meta‐regression identified 1052 genes for which the influence of the type of water stress treatment on the mean effect size ($\mu_{\mathrm{decracination}},\mu_{\mathrm{mannitol}},\mu_{\mathrm{water}-\mathrm{withholding}}$) was statistically significant (FDR < 0.05; Figure S1c and Table S3). Of these, a majority (736) have a larger expression change in response to deracination than to the other treatments (Figure S2a); it is likely that some of these reflect responses to wounding or other stresses rather than to water stress alone. The remaining 316 genes with a significant treatment effect respond more strongly to mannitol or water withholding than deracination; these appear to generally have opposing responses between mannitol and water withholding treatments (Figure S2e,f,h,i).

### Comparison of meta‐analysis with individual *t* test contrasts

**Figure 3:**
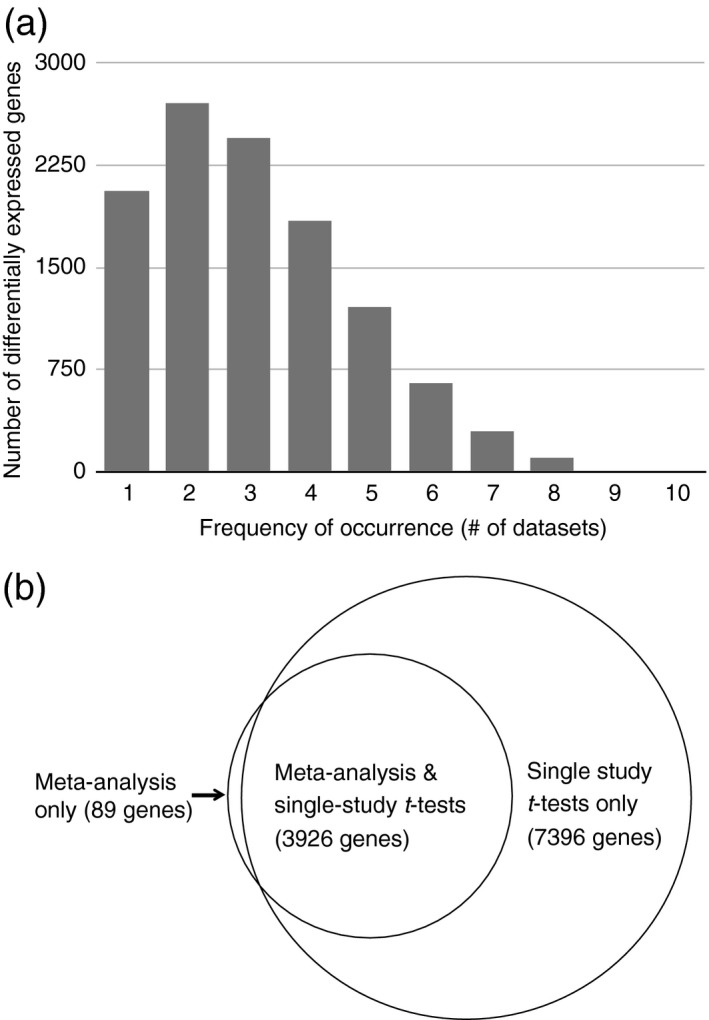
Comparison of genes identified via *t* tests in individual studies and genes identified in the meta‐analysis. (a) Distribution of the number of studies (contrasts) that each differentially expressed gene was identified in, according to individual *t* tests performed in each study. (b) Venn diagram showing the intersection between genes identified by a *t* test in at least one study, and those identified by the meta‐analysis.

We next sought to determine the extent that the 4015 genes identified by the meta‐analysis are concordant or complementary with results obtained by a simple summary of *t* test results from individual studies (Table 1; hereafter, the *t* test results for all of the genes in each individual study are referred to as a contrast). For each contrast, we performed moderated *t* test analyses (Smyth, 2004) of water stress‐treated plants versus control plants for each gene in each individual study, resulting in a list of DEGs (FDR < 0.05; Table 1). While there were 4015 DEGs identified by the meta‐analysis, a highly variable number of water stress‐responsive genes were identified in each contrast: ranging from zero to almost eight thousand DEGs (Table 1). In total, 35 036 genes (11 322 unique, Table S4) were identified as significantly differentially expressed in response to water stress in at least one contrast. Many genes (9264) were differentially expressed in two or more contrasts, while 2058 genes were identified in only a single contrast (Figure 3a). Two of the ten contrasts (GSE19700 and GSE10643) did not identify any DEGs; the analyses described below include only the eight contrasts for which DEGs were identified.

The eight single‐study contrasts were able to recapitulate 3926 of the DEGs identified by the meta‐analysis (Figure 3b and Table 1). The DEGs with the largest effect sizes according to the meta‐analysis were well represented in the lists of genes identified by single‐study contrasts (Table 2). The top 15 down‐regulated genes (ranked by magnitude of the effect size in the meta‐analysis) were discovered, on average, by six of the eight contrasts. The 15 genes most up‐regulated by water stress treatments according to the meta‐analysis, were also identified, on average, by six of the eight studies.

**Table 2:** Top five gene ontology differences between plant part‐specific genes detected by meta‐analysis and those detected by individual *t* test contrasts, according to *P* value (see Table S6 for complete results)

Category	Description	Gene ontology term	Relative frequency		Corrected *P* value
			Meta‐analysis	*t* tests	
Biological process	Photosynthesis	GO:0015979	7.1	3.6	5.3 × 10^−9^
	Photosynthesis, light reaction	GO:0019684	6.1	3	2.4 × 10^−9^
Cellular component	Photosystem	GO:0009521	1.6	0.3	4.5 × 10^−9^
	Chloroplast thylakoid membrane	GO:0009535	5.3	2.4	4.7 × 10^−9^
	Thylakoid part	GO:0044436	6.2	3	5.7 × 10^−9^

### Meta‐analysis identifies differentially expressed genes with small effect sizes

**Figure 4:**
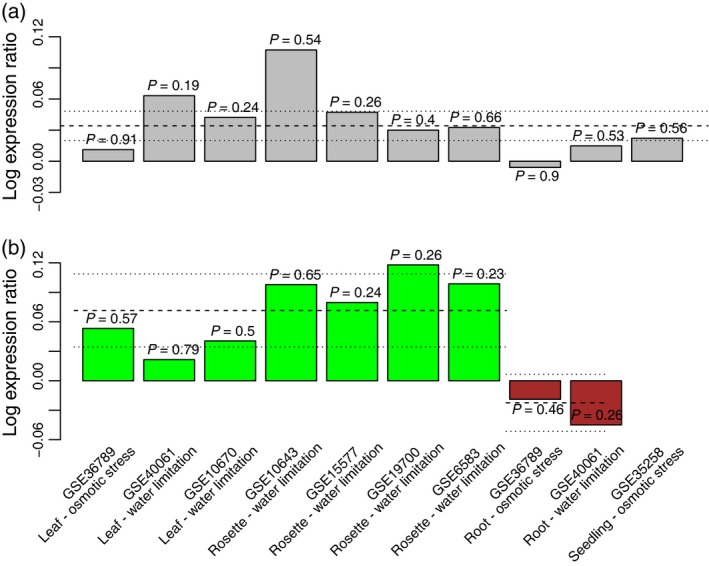
Examples of gene expression responses to drought that were detected by the meta‐analysis and meta‐regression, but not by the individual *t* test contrasts. (a) Expression response of *JAM3* across ten individual experiments, with *t* test *P* values for each experiment shown above each bar. $\hat{\mu }$ and its confidence interval from the meta‐analysis (FDR < 0.001) are indicated by dashed and dotted lines, respectively. (b) Expression responses of *EER5* across nine individual experiments. Plot as in (a), except that the results for experiments on shoots and roots are colored green and brown, respectively, and μ_shoots_ and $\mu_{\mathrm{roots}}$ and their confidence intervals from the meta‐regression (Q_M_ = 15.3, FDR = 0.002) are indicated by separate dashed/dotted lines for shoot and root experiments.

As discussed above, meta‐analyses have greater power to discover genes with small but consistent responses that may be missed in individual studies or found inconsistently among different studies due to their small responses. We found 89 genes that were identified only by the meta‐analysis and not discovered by the individual contrasts (Figure 3b). This collection of genes was enriched for genes with small effect sizes ($\hat{\mu }$). Seventy‐nine of the 89 genes (89%) had extremely small log expression ratios ($\hat{\mu }$ < 0.05, either induced or repressed) (Table S5). In comparison, only 14% of genes (544/4015) identified in the meta‐analysis had log expression ratios that were as small (<0.05). These 79 genes represent genes with small but consistent responses that the individual contrasts failed to identify, and their detection is potentially one of the advantages of performing meta‐analysis when summarizing results across studies. For example, *JAM3* (At4g16430) is slightly but consistently and significantly up‐regulated in response to water deficit according to the meta‐analysis ($\hat{\mu }$ = 0.03, FDR < 0.001; Table S5). While this gene is consistently up‐regulated in all but one of the individual contrasts, this up‐regulation was not significant according to the individual *t* tests (Figure 4a). The JAM3 transcription factor, along with JAM1 and JAM2, is a negative regulator of jasmonate responses. Previous work showed that *JAM1* and *JAM2* are induced by drought, but reported that *JAM3* expression is not significantly changed under any tested conditions (Sasaki‐Sekimoto *et al*., 2013). Our study suggests that this lack of significant change in any individual study may simply be due to its low magnitude of change, and the limited power available to detect low‐magnitude changes in any single study. These genes with small but highly consistent responses may be important for understanding the transcriptional biology of stress responses. However, we cannot say how important they are until careful empirical studies are performed to evaluate their contributions.

### Meta‐regression identifies water stress‐responsive genes moderated by plant part or treatment

Individual studies may have different results for a number of reasons. It is important to identify differences between responses in specific plant parts, and between different experimental treatments, unambiguously and quantitatively. We used meta‐regression to summarize the magnitude and significance of these covariates across studies. We compared the 1360 genes identified as having plant part‐specific expression by the meta‐regression with the 4173 genes identified as having plant part‐specific expression by the *t* test contrasts (by comparing *t* test results, i.e. significant versus non‐significant in different contrasts). Of the latter 4173 genes, only 13% (550) were also identified as plant part‐specific by the meta‐regression. These 550 common genes represent only 40% of the 1360 genes identified as differing among plant parts according to the meta‐regression. We next used a statistical test to assess whether there are biological differences between the sets of genes identified as plant part‐specific by meta‐regression versus by comparison among *t* test contrasts (Sánchez *et al*., 2007; Salicrú *et al*., 2011). In comparison to the meta‐regression, the *t* test approach missed genes involved in 51 gene ontology terms, including tissue‐specific processes such as photosynthesis and light harvesting, with components of both photosystem I and II stabilization, and with protein products localized to the thylakoid (Table 2 and Table S6). In contrast to these 51 gene ontology terms, only three terms occur at a higher frequency among genes categorized as plant part‐specific by comparing *t* tests than among genes identified by the meta‐regression (RNA modification and processing, cytosol localization), none of which are related to plant part‐specific responses.

We performed a similar analysis comparing the 1052 genes differentially expressed in response to the experimental method by the meta‐regression versus the 4479 identified by comparing the individual *t* tests. Of the latter 4479 genes, only 6% (274) were also identified as method‐specific by the meta‐regression. These 274 common genes represent only 26% of the 1052 genes identified as moderated by experimental method according to the meta‐regression. Again, we looked at gene ontology profiles to evaluate these differences. In comparison with the meta‐regression, the *t* test approach shows a significantly lower frequency of genes in abiotic stress‐related gene ontology terms, such as response to wounding, osmotic stress and water deprivation (Table S7). These responses make biological sense because of the differences between the methods used in the analyzed experiments: mannitol treatment results in osmotic stress and deracination results in a wounding response. In contrast, the gene ontology terms found at higher frequency among genes identified in the *t* test comparison do not appear to be related to method‐specific functions.

At the extreme, we found genes identified by the meta‐regression that were not identified by even a single *t* test contrast (33 genes for plant part and 56 genes for treatment type; Tables S8 and S9). For example, *EER5* (At2g19560), which encodes a transcription factor involved in ethylene signaling, shows significant differential responses in roots and shoots across studies in response to water stress (Figure 4b; Q_M_ = 15.3, FDR = 0.002). The gene is significantly up‐regulated in shoots (*P *<* *0.001) and down‐regulated in roots (non‐significant). These responses are consistent across all individual studies; however, this effect was not significant in any individual study. Ethylene has been shown to play a central role in coordinating growth and survival in response to diverse challenges, including drought (Yoo *et al*., 2009). Recent work in an Arabidopsis *eer5* mutant suggests that ethylene regulation is complex, potentially including EER5‐dependent transcription of genes responsible for inhibiting or re‐setting ethylene responses (Christians *et al*., 2008). Previous transcriptional profiling resulted in identification of a few ethylene biosynthesis genes, suggesting a link between ethylene and drought‐stress signaling pathways in Arabidopsis (Seki *et al*., 2002), but *EER5* has not previously been implicated. Here, the statistical power afforded by the meta‐regression resulted in the discovery of a tissue specific‐action of this gene in the water stress response. While the magnitude of this response is low, its consistent pattern when all studies were considered together suggests that it is biologically meaningful.

### Meta‐analysis excludes idiosyncratic responses associated with individual studies and treatments

The single‐study *t* tests were largely able to recapitulate the water stress‐responsive genes identified by the meta‐analysis (Figure 3b). Most of the genes that were identified by all eight individual studies (88%, 90/102) were also identified by the meta‐analysis. In contrast, few (11%) of the water stress‐responsive genes that were identified by *t* test in only a single study were identified as differentially expressed by the meta‐analysis. The DEGs identified by *t* test in the deracination study (GSE6583) that were not identified by the meta‐analysis were enriched for the gene ontology term for responses to wounding (GO:0009611; FDR < 0.001, Table S10). This suggests that meta‐analysis may exclude idiosyncratic responses associated with particular treatments, such as wounding, as opposed to the conserved water stress response. The statistical (and biological) reason for this is that these genes vary considerably in their responses among studies due to treatment differences, as well as unidentified batch effects. In addition, high variance among biological replicates within studies may be caused by inconsistencies among biological replicates or low numbers of biological replicates. By excluding such results with large confidence intervals meta‐analysis is able to exclude idiosyncratic responses.

## Discussion

The massive amount of publicly available transcriptome data affords the opportunity to identify broad patterns in transcriptome remodeling that are not discernible from the small number of samples typically included in a single study. A number of approaches have been proposed to synthesize the results of multiple studies. Meta‐analysis has been used in other fields to synthesize transcriptomic data, and should also be more widely implemented for plant differential response data. Shaik and Ramakrishna (2013) and Zaag *et al*. (2015) synthesized DEG microarray studies on biotic and abiotic stress responses in Arabidopsis, and identified genes that were differentially expressed in response to each stress. Although these approaches were each shown to be effective in various ways, for example in assisting with functional annotations and visualizing co‐expression relationships, the disadvantages of these approaches are that they do not weight studies based on precision (i.e. variance), distinguish between sampling variance within studies and between‐study variance, or estimate variance components due to covariates (stress type, method, plant part).

We used inverse‐variance weighted meta‐analysis and meta‐regression to identify sources of heterogeneity in study outcomes. Comparison of the meta‐analysis and meta‐regression results with results from individual *t* test contrasts was informative. We found that most of the genes discovered by meta‐analysis were congruent with those identified by the moderated *t* test contrasts. A number of small but highly consistent differential responses were identified only by the meta‐analysis. Interestingly, in some cases, individual studies may contain useful information that was previously not apparent. For example, while two of the ten studies that we included identified no significant DEGs when examined individually (possibly due to low statistical power), they probably contain useful information when statistically combined with other studies using meta‐analysis.

The results of the meta‐regression also revealed some results that were not apparent using moderated *t* test contrasts. Most strikingly, a significant fraction of genes involved in photosynthesis that are differentially expressed between roots and shoots were missed. In addition, specific genes involved in other functions, such as *EER5*, which encodes a regulator of ethylene in shoots, were found to be differentially expressed in the meta‐regression, but were not identified in even a single *t* test. These missed genes highlight the limitations of vote‐counting analyses that compare published lists of DEGs, which tend to identify only a small number of the most consistent responses across multiple studies, probably as a result of low statistical power. For example, a comparison of three microarray studies of the response of Arabidopsis to water stress identified only 27 common water stress‐induced genes, representing just over 3% of the 806 water stress‐induced genes identified by the individual studies (Bray, 2004). A systematic investigation of microarray studies, undertaken by the MicroArray Quality Control (MAQC) project, showed that a combination of fold change ranking and careful selection of *P *value cutoffs when identifying DEGs greatly increased the reproducibility of microarray studies within and between laboratories, and across platforms (Shi *et al*., 2008). In the present study, we applied the MAQC approach to obtain gene lists that are more comparable between studies than would be achieved by comparing results obtained from study‐specific analysis parameters. Even so, any vote‐counting approach may still be subject to idiosyncratic responses, such as those due to methodological differences among studies. Indeed, we found that the number and identity of DEGs identified by *t* tests was highly variable across studies (Table 1). Some of these differences may be explained by the true biological variation underlying the complex response of Arabidopsis to environmental change. Differences in experimental methods (e.g. the mode, duration and severity of treatment), the developmental stage of the plants, or the time of day at which the samples were collected (Wilkins *et al*., 2009, 2010) may influence the induced transcriptome remodeling. Genes detected in the deracination experiment, but not in the meta‐analysis, were enriched in wounding‐related gene ontology terms, suggesting that meta‐regression distinguished conserved water stress‐responsive genes but excluded those associated with a particular treatment.

Adams *et al*. (2008) used meta‐analysis to integrate microarray studies on differential gene expression in honey bee brains at different stages in the maturation of the bees, and compared their results with the results of conventional individual study analyses. Similar to our findings, they reported that meta‐analysis was able to identify genes with consistent overall expression patterns, and also rejected genes with inconsistent expression across studies. They also found that comparing lists of genes identified in individual studies failed to discover genes with consistent expression across studies that were below the selected significance threshold.

### Meta‐analysis reveals consistent responses among genes involved in abscisic acid signaling

Many of the water stress‐responsive genes identified in the meta‐analysis are canonical regulators of the water stress response in *A. thaliana*, and include a number of genes in the abscisic acid (ABA) signaling pathway. ABA is a well‐characterized plant hormone that regulates plant responses to drought and water stress (Fujita *et al*., 2011). It is produced in the roots of drought and water‐stressed plants and is transported to the leaves, where it regulates stomatal aperture to prevent continued water loss (Schachtman and Goodger, 2008). The minimal ABA signaling pathway comprises members of the regulatory component of ABA receptor (RCAR/PYR/PYL) family, type 2C protein phosphatases (PP2C), and members of the SNF1‐related kinase 2 (SnRK2) family (Sheard and Zheng, 2009).

Our meta‐analysis identified changes in members of all three of these signaling pathway families (Table S11). Importantly, the meta‐analysis permits us to quantitatively summarize the relative effect size of each component of this pathway across numerous studies. The *HAI2*/*AIP1* gene (*highly ABA‐induced PP2C gene 2*; At1g07430) had the largest effect size ($\hat{\mu }$ = 0.73) in the meta‐analysis. It was also one of the top 100 DEGs in seven of the eight single‐study *t* tests, but its position differed substantially between studies. *HAI2*/*AIP1* encodes a protein that acts as a negative regulator of osmoregulatory solute accumulation during drought and water stress (Bhaskara *et al*., 2012) and as a positive regulator of ABA signaling (Lim *et al*., 2012). *RCAR3* (At5g53160), a gene that encodes a binding partner of *HAI2*/*AIP1*, was significantly down‐regulated in response to water stress in the meta‐analysis ($\hat{\mu }$ = −0.10). *SnRK2–6* (At4g33950), a gene that encodes a calcium‐independent ABA‐activated protein kinase with a role in ABA‐mediated regulation of stomatal aperture, was up‐regulated in response to water stress in the meta‐analysis ($\hat{\mu }$ = 0.15) and in five of the single‐study *t* tests.

## Conclusions

Formal statistical methods for synthesizing the results of gene expression responses from a number of studies are increasingly being implemented in various research fields. We found that, while the results of moderated *t* test syntheses agreed in large part with the results of our meta‐analysis, formal weighted meta‐analysis was able to discover additional responses that were not identified by conventional approaches. Meta‐regression provided additional information on responses that are attributable to use of shoots versus roots and to the methodology used that may be missed using other approaches for synthesis of results. Identification of gene responses across studies, and of the sources of heterogeneity in responses, may be accurately addressed using meta‐analysis and meta‐regression, and the results may provide additional biologically meaningful information. With some development, these approaches may also be adapted for other types of transcriptomic data, such as RNA‐seq data.

## Experimental procedures

### Gene expression data

Data sets published in the National Center for Biotechnology Information Gene Expression Omnibus (GEO) at 17 October 2012 were reviewed for inclusion in this study. The basic criteria for inclusion were as follows: (i) studies used the ATH1 Genome Array, platform GPL198 (699 studies), (ii) study titles included at least one of the following terms: drought, water stress, water potential, osmotic (33 studies), (iii) studies included paired treated and untreated samples, and (iv) samples were derived from wild‐type Col–0 plants to provide as consistent a data set as possible. These criteria resulted in a total of ten GEO series comprising 56 arrays (Table 1). For studies that included time series data, a single daytime point was selected; the other time points were excluded.

### Pre‐processing

The raw expression data files (CEL files) for the study samples were downloaded from GEO. CEL files for each study were pre‐processed separately using GCRMA (Wu *et al*., 2004). Probe sets that did not satisfy the following standard criteria were excluded from downstream analyses: (i) expression levels had to have a minimum log_2_ fluorescence intensity of 100 in at least 10% of samples, and (ii) expression levels were required to have a minimum interquartile log_2_ range of 0.5 across all arrays. A total of 11 985 probe sets, of the 24 445 probe sets on the array, met these criteria and were included in the meta‐analysis. By removing genes with low levels of expression and with little variation, the number of statistical tests required is greatly reduced. However, a trade‐off for this reduced number of multiple tests is the potential for false negatives.

Analysis of the microarray data was performed using Bioconductor suite 2.11 (Gentleman *et al*., 2004) in R 2.14.2 (R Development Core Team, 2009; http://www.R-project.org) using the affy 1.36.1 package (Gautier *et al*., 2004).

All statistical tests (*P* values) were corrected for multiple comparisons using the Benjamini–Hochberg false discovery rate correction (Benjamini and Hochberg, 1995) using a step‐up procedure (Wright, 1992), as implemented in the p.adjust function of R. This approach adjusts *P* values so that the FDR is at the desired level of α (here 0.05; i.e. the expected proportion of false discoveries is 5%).

### Meta‐analysis

A separate meta‐analysis was performed on the data for each included gene that met the criteria above, to synthesize differential responses across the different studies. We followed standard established meta‐analysis methodology (Viechtbauer, 2010; Borenstein *et al*., 2011; Mengersen and Schmid, 2013; Rosenberg *et al*., 2013). We calculated effect sizes for each gene (probe set) for each study as the log expression ratio (ln*R*; Hedges *et al*., 1999) for differential expression:

(1) $$\mathrm{In}\;\;R_{i}=\text{ln}\left(\frac{\bar{Y1}}{\bar{Y2}}\right)=\text{ln}\bar{Y1}-\text{ln}\bar{Y2}$$

and its approximated variance:

(2) $$v_{i}=\frac{s_{1}^{2}}{n_{1}{\bar{Y}}_{1}^{2}}+\frac{s_{2}^{2}}{n_{2}{\bar{Y}}_{2}^{2}}$$

for the *i*th study, where ${\bar{Y}}_{1}$ is the mean response to the experimental (water stress) treatment for a gene for the biological replicates within an individual study and ${\bar{Y}}_{2}$ is the mean response for the control (non‐stress) treatment, $s_{1}^{2}\text{and}s_{2}^{2}$ are their respective variances, and *n*_1_ and *n*_2_ are the respective sample sizes (number of biological replicates).

To combine the studies, we used a random‐effects model in which studies are weighted by the sum of the true variation among studies and sampling variation within studies:

(3) $$w_{i}=\frac{1}{v_{i}+\tau^{2}}$$

where *v*_*i*_ is defined above and τ^2^ is the between‐study variance.

The effect sizes were combined across studies to give a weighted mean effect size across *K* studies:

(4) $$\hat{\mu }=\frac{\sum_{i=1}^{K}w_{i}{\hat{\theta }}_{i}}{\sum_{i=1}^{K}w_{i}}$$

and the variance of $\hat{\mu }$:

(5) $$s_{\hat{\mu }}^{2}=\frac{1}{\sum_{i=1}^{K}w_{i}}$$

where $\hat{\theta }$ is the effect size of each gene for each study, and *w*_*i*_ is the corresponding weight for that study, and the 95% confidence interval around $\hat{\mu }$ is:

(6) $$\text{CI}=\hat{\mu }\pm s_{\hat{\mu }}t_{\frac{\alpha }{2}\left[K-1\right]}$$

where *t* is Student's *t* distribution with *K*‐1 degrees of freedom at an α level of 0.05. The confidence intervals and test statistics (*z* value, and its associated *P* value) of the individual coefficients in the model (i.e. whether the gene expression response is significantly different from zero) are based on the normal distribution. The *P* values across genes were FDR‐adjusted, as described above. The random‐effects model was fitted using a restricted maximum‐likelihood estimator.

Calculations for Eqns (3–6) were performed using the R package metafor version 1.7 (Viechtbauer, 2010). As discussed in Lajeunesse (2015), bias may be introduced into the variance calculation when means are near zero because of our relatively small sample sizes. However, while small sample size corrections have been suggested (Hedges *et al*., 1999, 2010; Moreno *et al*., 2012; Lajeunesse, 2015), they have not yet been adopted in the literature or implemented in software, and so we applied the widely used formulation here. More detailed explanations and assumptions of the random‐effects model are discussed elsewhere (Hedges and Olkin, 1985; Borenstein *et al*., 2011; Mengersen *et al*., 2013; Rosenberg *et al*., 2013; Gurevitch and Nakagawa, 2015).

### Meta‐regression

Meta‐regressions were performed separately to evaluate the contribution of two covariates to expression responses for each gene: plant part and water stress method. First, we assessed the effect of plant part on expression. We identified plant part as roots or shoots, where shoots are above‐ground tissue, including rosettes and leaves. We excluded the study GSE35258, which sampled seedlings, from the analysis. For studies in which responses for different plant parts were assessed, we included the outcomes reported for different plant parts as if they were independent studies. While this is not a conservative approach, the data available were insufficient to include a complete statistical model, and the trade‐off was preserving this valuable information at the cost of violating the assumption of independence among outcomes. We then included plant part as a covariate:

(7) $$y_{i}=\beta_{0}+\beta_{1}X_{i}+\sigma_{i}+e_{i}$$

where β_0_ is the intercept (the mean true effect when the moderator variable is equal to zero), β_1_ is the regression coefficient for plant part, *X*_*i*1_ is the categorical dummy value of the covariate (roots or shoots) for the *i*th study, σ_*i*_ is the random‐effects error term (the residual heterogeneity), and *e*_*i*_ is the independent sampling error. In addition, the meta‐regression was performed without an intercept so that the model coefficients may be interpreted as mean effect size estimates. To estimate the random effects variance, we used the DerSimonian–Laird estimator (DerSimonian and Laird 1986; Raudenbush, 2009). We are most interested in the values of the coefficient β_1_ for roots and shoots, as well as their confidence intervals and test statistics. The test statistics of the individual coefficients in the model (and the corresponding confidence intervals) are based on the normal distribution.

In order to determine whether the plant part covariate was significant for a particular gene, we performed a heterogeneity test and calculated Q_M_, the *Q*‐statistic for the model, which tests whether at least one of the regression coefficients is different from zero (Viechtbauer, 2010; Borenstein *et al*., 2011), as well as its *P* value, where *Q* is χ^2^‐distributed with *m − *1 degrees of freedom (*m* being the number of coefficients tested). We adjusted *P* using an FDR correction, as described above. A more complete description of the meta‐regression methods is available in the literature (Thompson and Higgins, 2002; Hedges *et al*., 2010; Schwarzer *et al*., 2015).

Second, we evaluated the effect on differential expression of the method of imposing water stress. Methods used to impose water stress were water withholding, deracination, and osmotic stress (induced by mannitol). We excluded the study GSE35258, in which polyethylene glycol was used to induce water stress, from the analysis. The influence of the method on differential expression was treated as a covariate in a random‐effects model, exactly as described above for plant part (Eqn 7).

The meta‐regressions on plant part and water stress method were performed separately because of sampling limitations in the current dataset (i.e. to include seedlings when assessing the effect of method, but not when assessing roots versus shoots, and to exclude polyethylene glycol as a method when assessing method), although meta‐regression models may also be used to simultaneously evaluate multiple covariates.

### *t* tests for individual contrasts

The limma 3.13.4 Bioconductor package was used to identify DEGs in individual studies (i.e. individual contrasts) (Smyth, 2005). In each case, water deficit‐treated samples were contrasted with their paired controls to identify significant DEGs. Each contrast was conducted as a moderated *t* test, to identify DEGs between the water‐stressed and control samples in a single tissue in a given study. A moderated *t *test may be interpreted just as an ordinary *t *statistic, the only difference being that the standard errors have been moderated across genes, which has been shown to improve performance on microarray data (Smyth, 2004). No other effects (e.g. study, tissue) were evaluated in this analysis. The pairwise *t* tests used the same pre‐processed data as used in the meta‐analysis.

Gene ontology enrichment analysis was performed by singular enrichment analysis using agriGO (Du *et al*., 2010), with all genes on the Affymetrix ATH1 Genome Array (GPL198) as background. Significance of enrichment was determined using a hypergeometric test (FDR‐corrected *P* value <0.05). Gene ontology profile analysis was used to compare gene lists obtained from meta‐analysis to lists obtained from individual contrasts in the R package goProfiles version 1.30 (Du *et al*., 2010; Sánchez *et al*., 2013). The significance of profile differences in annotation frequencies were tested for each gene ontology term between levels 2 and 15 for molecular function, biological process and cellular component, using Fisher's exact test. We then applied an FDR correction to the complete set of tests, following the procedure described above.

## Supporting information


**Figure S1.** Statistical properties of meta‐analysis and meta‐regression models.

**Figure S2.** Plot illustrating the effect of the experimental method on which genes respond to water stress, according to the meta‐regression.

**Table S1.** Arabidopsis genes with a significant differential expression response to water limitation, according to our meta‐analysis of ten studies.

**Table S2.** Genes with expression responses to stress that are significantly moderated by plant part (shoots versus roots), according to the meta‐regression.

**Table S3.** Genes identified by the meta‐regression with a significant effect of the type of water stress treatment on the expression response.

**Table S4.** Differentially expressed genes in each study (contrast), according to individual *t* tests.

**Table S5.** Genes identified only by the meta‐analysis and not discovered by the individual *t* test contrasts.

**Table S6.** Comparison of frequencies of gene ontology terms for genes where the plant part moderates their expression, identified either by meta‐regression or by comparison of *t* test results across experiments.

**Table S7.** Comparison of frequencies of gene ontology terms for genes where the experimental method type moderates their expression, identified either by meta‐regression or by comparison of *t* test results across experiments.

**Table S8.** Genes identified by the meta‐regression as having expression responses to water stress that are moderated by plant part, but which were not identified by even a single *t* test.

**Table S9.** Genes identified by the meta‐regression as having expression responses to water stress that are moderated by the type of experimental method, but which were not identified by even a single *t* test.

**Table S10.** Results of gene ontology enrichment analysis for genes with a significant gene expression effect only in a single deracination study.

**Table S11.** Abscisic acid pathway‐related genes identified by the meta‐analysis.
