# Supplementary material for: Meta‐analysis and meta‐regression of transcriptomic responses to water stress in Arabidopsis
Source: Plant J. 2016 Feb 12;85(4):548–60. doi: 10.1111/tpj.13124 (PMC4815425; doi:10.1111/tpj.13124)
Supplement: Supplementary file 1 — Figure S1. Statistical properties of meta‐analysis and meta‐regression models. [file TPJ-85-548-s001.docx]

**Fig. S1.** Statistical properties of meta-analysis and meta-regression models. (a) The distribution of effect sizes ($\hat{\mu}$), P-values, and Q-values are provided for the grand means meta-analysis. (b) The distribution of effect sizes for genes in experiments testing different plant parts (tissues): shoots (leaves) and roots. The distribution of QMp values (significance of the effect of plant part) are also shown. (c) The distribution of effect sizes for genes from experiments where water deficit was caused by different treatments (deracination, water withholding, or mannitol). The distribution of QMp values (significance of the effect of treatment method) are also shown.

**
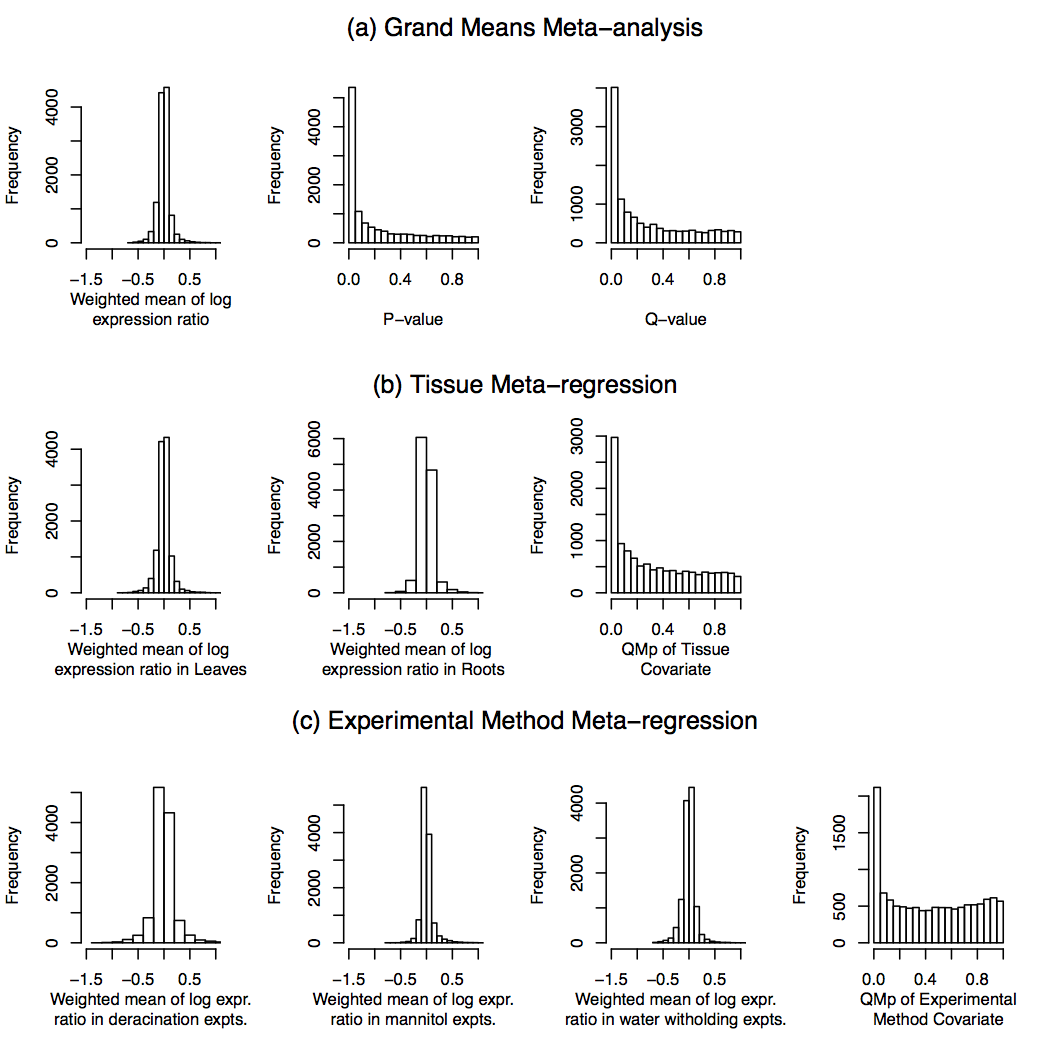
**
