# Supplementary material for: Meta‐analysis and meta‐regression of transcriptomic responses to water stress in Arabidopsis
Source: Plant J. 2016 Feb 12;85(4):548–60. doi: 10.1111/tpj.13124 (PMC4815425; doi:10.1111/tpj.13124)
Supplement: Supplementary file 2 — Figure S2. Plot illustrating the effect of the experimental method on which genes respond to water stress, according to the meta‐regression. [file TPJ-85-548-s002.docx]

**Fig. S2.** Genes with a significant effect of experimental method (FDR corrected QMp < 0.05) were separated into three unique groups: genes that have their maximum expression change in response to deracination (first row; blue), genes that have their maximum expression change in response to mannitol (second row; green), and genes that have their maximum expression change in response to water withholding (third row; gold color). Within each row of plots, genes are sorted by its largest effect (max[$\mu_{deracination},\mu_{mannitol},\mu_{water-witholding}$], either up or down). Each gene is shown with a dot ($\mu_{deracination},\mu_{mannitol},\mu_{water-witholding}$) and horizontal line (95% CI).
