# Supplementary material for: Meta‐analysis and meta‐regression of transcriptomic responses to water stress in Arabidopsis
Source: Plant J. 2016 Feb 12;85(4):548–60. doi: 10.1111/tpj.13124 (PMC4815425; doi:10.1111/tpj.13124)
Supplement: Supplementary file 8 — Table S6. Comparison of frequencies of gene ontology terms for genes where the plant part moderates their expression, identified either by meta‐regression or by comparison of t test results across experiments. [file TPJ-85-548-s008.docx]

**Table S6.** Comparison of gene ontology frequencies of genes where the plant part (shoots vs. roots) moderates their expression, identified either by meta-regression or by comparison of t-test results across experiments. Class: BP=biological process, CC=cellular component. There were no significant differences between meta-analysis and t-test contrast frequencies in the molecular function category. An FDR corrected p-value based on Fisher’s exact test indicates the level of significant difference between the two frequencies. GO terms with higher frequencies in the meta-analysis are found at the top of the list, and GO terms with higher frequencies in t-tests are found at the bottom of the list.

| **class** | **GOID** | **Description** | **Meta-analysis frequency** | **t-test contrast frequency** | **Corrected p** |
| --- | --- | --- | --- | --- | --- |
| BP | GO:0000096 | sulfur amino acid metabolic process | 3.5 | 2.3 | 0.00023534 |
| BP | GO:0000097 | sulfur amino acid biosynthetic process | 3.5 | 2 | 3.39083E-05 |
| BP | GO:0006364 | rRNA processing | 3.9 | 2.4 | 0.000356764 |
| BP | GO:0006470 | protein dephosphorylation | 2.7 | 1.5 | 0.00012027 |
| BP | GO:0006520 | cellular amino acid metabolic process | 7.5 | 4.8 | 5.01119E-06 |
| BP | GO:0006534 | cysteine metabolic process | 3.2 | 1.3 | 2.54604E-07 |
| BP | GO:0006787 | porphyrin-containing compound catabolic process | 1.4 | 0.4 | 2.42328E-05 |
| BP | GO:0006873 | cellular ion homeostasis | 2 | 0.9 | 0.000394855 |
| BP | GO:0008652 | cellular amino acid biosynthetic process | 5.2 | 3.4 | 3.67461E-05 |
| BP | GO:0009069 | serine family amino acid metabolic process | 3.6 | 1.9 | 7.65402E-06 |
| BP | GO:0009070 | serine family amino acid biosynthetic process | 3.4 | 1.4 | 1.98969E-07 |
| CC | GO:0009521 | Photosystem | 1.6 | 0.3 | 4.51382E-08 |
| CC | GO:0009522 | photosystem I | 0.8 | 0.1 | 7.49441E-07 |
| CC | GO:0009534 | chloroplast thylakoid | 6.7 | 3.6 | 1.01445E-06 |
| CC | GO:0009535 | chloroplast thylakoid membrane | 5.3 | 2.4 | 4.67105E-08 |
| CC | GO:0009538 | photosystem I reaction center | 0.4 | 0 | 0.000127136 |
| CC | GO:0009579 | Thylakoid | 7.5 | 4.2 | 2.07851E-07 |
| BP | GO:0009648 | Photoperiodism | 2.5 | 0.9 | 0.000235231 |
| BP | GO:0009765 | photosynthesis, light harvesting | 0.8 | 0.2 | 0.000182456 |
| BP | GO:0010109 | regulation of photosynthesis | 1.1 | 0.2 | 0.000188881 |
| BP | GO:0010207 | photosystem II assembly | 3.1 | 1.7 | 0.000340144 |
| BP | GO:0015979 | Photosynthesis | 7.1 | 3.6 | 5.32166E-09 |
| BP | GO:0015996 | chlorophyll catabolic process | 1.4 | 0.4 | 1.42928E-05 |
| BP | GO:0016072 | rRNA metabolic process | 3.9 | 2.5 | 0.0003831 |
| BP | GO:0016311 | Dephosphorylation | 3.2 | 1.7 | 0.000154233 |
| BP | GO:0019344 | cysteine biosynthetic process | 3.2 | 1.3 | 1.80196E-07 |
| BP | GO:0019439 | aromatic compound catabolic process | 4 | 1.9 | 3.99005E-05 |
| BP | GO:0019684 | photosynthesis, light reaction | 6.1 | 3 | 2.39468E-08 |
| BP | GO:0022900 | electron transport chain | 2 | 0.9 | 0.000483585 |
| CC | GO:0031976 | plastid thylakoid | 6.7 | 3.6 | 1.01445E-06 |
| CC | GO:0031984 | organelle subcompartment | 6.7 | 3.6 | 1.01445E-06 |
| BP | GO:0033015 | tetrapyrrole catabolic process | 1.4 | 0.4 | 2.42328E-05 |
| CC | GO:0034357 | photosynthetic membrane | 5.4 | 2.6 | 9.52893E-08 |
| BP | GO:0035303 | regulation of dephosphorylation | 2.4 | 1.2 | 0.000171661 |
| BP | GO:0035304 | regulation of protein dephosphorylation | 2.4 | 1.1 | 5.30335E-05 |
| CC | GO:0042651 | thylakoid membrane | 5.4 | 2.6 | 2.74937E-07 |
| BP | GO:0044270 | cellular nitrogen compound catabolic process | 4.1 | 1.9 | 2.26503E-05 |
| BP | GO:0044272 | sulfur compound biosynthetic process | 5.1 | 3.6 | 0.000319701 |
| CC | GO:0044436 | thylakoid part | 6.2 | 3 | 5.71969E-08 |
| BP | GO:0046149 | pigment catabolic process | 1.4 | 0.4 | 2.42328E-05 |
| BP | GO:0046700 | heterocycle catabolic process | 4.1 | 1.8 | 1.03979E-05 |
| BP | GO:0048573 | photoperiodism, flowering | 2.5 | 0.9 | 0.000235231 |
| BP | GO:0048878 | chemical homeostasis | 2.7 | 1.4 | 0.000171656 |
| BP | GO:0051187 | cofactor catabolic process | 1.4 | 0.4 | 3.98071E-05 |
| CC | GO:0055035 | plastid thylakoid membrane | 5.3 | 2.4 | 5.80649E-08 |
| BP | GO:0055082 | cellular chemical homeostasis | 2.1 | 1 | 0.000448033 |
| BP | GO:1901361 | organic cyclic compound catabolic process | 4.1 | 2 | 3.39083E-05 |
| BP | GO:1901564 | organonitrogen compound metabolic process | 15.3 | 11.8 | 5.3858E-05 |
| BP | GO:1901565 | organonitrogen compound catabolic process | 3.8 | 1.9 | 6.31831E-05 |
| BP | GO:1901605 | alpha-amino acid metabolic process | 5.8 | 3.7 | 7.49623E-05 |
| BP | GO:1901607 | alpha-amino acid biosynthetic process | 4.3 | 2.7 | 0.000115424 |
| BP | GO:0009451 | RNA modification | 1.7 | 3.6 | 0.000170499 |
| BP | GO:0006396 | RNA processing | 1.6 | 4.2 | 7.35091E-06 |
| CC | GO:0005829 | Cytosol | 8.5 | 11.5 | 0.000389985 |
