# Supplementary material for: Meta‐analysis and meta‐regression of transcriptomic responses to water stress in Arabidopsis
Source: Plant J. 2016 Feb 12;85(4):548–60. doi: 10.1111/tpj.13124 (PMC4815425; doi:10.1111/tpj.13124)
Supplement: Supplementary file 10 — Table S8. Genes identified by the meta‐regression as having expression responses to water stress that are moderated by plant part, but which were not identified by even a single t test. [file TPJ-85-548-s010.docx]

**Table S8.** Genes identified by the meta-regression as having expression responses to water stress that are moderated byorgan (roots vs. shoots), but which were not identified by even a single t-test. The effect sizes ($\mu_{roots},\mu_{shoots}$) and significance estimates calculated by the meta-regression for each organ are provided.

| **ID** | **Gene Symbol** | **AGI_TAIR** | **$\mu_{shoots}$** | **$\mu_{roots}$** | **p_shoots_** | **p_roots_** | **QM** | **QMp** | **fdr (QMp)** |
| --- | --- | --- | --- | --- | --- | --- | --- | --- | --- |
| 245263_at |  | AT4G17740 | -0.0769 | 0.0082 | 0.0000 | 0.7506 | 7.7306 | 0.0054 | 0.0485 |
| 246998_at |  | AT5G67370 | 0.0023 | -0.2199 | 0.8561 | 0.0050 | 7.8483 | 0.0051 | 0.0461 |
| 248379_at | PBS2 | AT5G51700 | 0.0370 | -0.0127 | 0.0007 | 0.2341 | 10.5828 | 0.0011 | 0.0159 |
| 248421_at |  | AT5G51510 | 0.0248 | -0.0050 | 0.0000 | 0.3034 | 18.9020 | 0.0000 | 0.0005 |
| 249192_at |  | AT5G42470 | 0.0659 | -0.0176 | 0.0000 | 0.4890 | 8.2222 | 0.0041 | 0.0398 |
| 249385_at | ATTRX2 | AT5G39950 | 0.0187 | -0.0169 | 0.0018 | 0.0324 | 12.8794 | 0.0003 | 0.0062 |
| 249398_at |  | AT5G40250 | 0.0313 | -0.0163 | 0.0245 | 0.0913 | 7.8967 | 0.0050 | 0.0453 |
| 250334_at |  | AT5G11770 | 0.0309 | -0.0210 | 0.0006 | 0.0781 | 12.1214 | 0.0005 | 0.0086 |
| 252012_at |  | AT3G52730 | -0.0137 | 0.0072 | 0.0003 | 0.1294 | 11.7488 | 0.0006 | 0.0099 |
| 252142_at |  | AT3G51120 | 0.0427 | -0.0232 | 0.0023 | 0.1670 | 9.0974 | 0.0026 | 0.0290 |
| 253204_at | AGB1 | AT4G34460 | 0.0376 | -0.0145 | 0.0002 | 0.3096 | 8.8258 | 0.0030 | 0.0320 |
| 253223_at | APX3 | AT4G35000 | -0.0115 | 0.0236 | 0.0203 | 0.0127 | 10.7960 | 0.0010 | 0.0147 |
| 253238_at |  | AT4G34480 | -0.0204 | 0.0387 | 0.1794 | 0.0000 | 12.1384 | 0.0005 | 0.0085 |
| 254181_at |  | AT4G23940 | -0.0783 | 0.0350 | 0.0000 | 0.0074 | 26.8147 | 0.0000 | 0.0000 |
| 254477_at |  | AT4G20380 | 0.0443 | -0.0018 | 0.0000 | 0.8518 | 14.1299 | 0.0002 | 0.0036 |
| 256682_at | LTA3 | AT3G52200 | 0.0302 | -0.0176 | 0.0000 | 0.0442 | 19.5774 | 0.0000 | 0.0004 |
| 256910_at |  | AT3G24080 | -0.0909 | 0.0309 | 0.0000 | 0.1664 | 18.8686 | 0.0000 | 0.0005 |
| 258171_at |  | AT3G21610 | 0.0545 | -0.0123 | 0.0000 | 0.3545 | 16.2797 | 0.0001 | 0.0015 |
| 258186_s_at |  | AT1G48490 AT3G17850 | 0.0446 | -0.0216 | 0.0006 | 0.1751 | 10.3977 | 0.0013 | 0.0171 |
| 258511_at |  | AT3G06590 | 0.0316 | -0.0333 | 0.0000 | 0.0011 | 28.8394 | 0.0000 | 0.0000 |
| 258522_at |  | AT3G06660 | -0.0875 | -0.0130 | 0.0000 | 0.4699 | 12.6880 | 0.0004 | 0.0067 |
| 260398_at | APUM23 | AT1G72320 | -0.0918 | -0.0287 | 0.0000 | 0.0589 | 11.0974 | 0.0009 | 0.0130 |
| 260785_at |  | AT1G06200 | 0.0249 | -0.0161 | 0.0003 | 0.0106 | 19.1785 | 0.0000 | 0.0004 |
| 261426_at |  | AT1G18680 | 0.0359 | -0.0349 | 0.0001 | 0.0210 | 16.2496 | 0.0001 | 0.0015 |
| 261940_at |  | AT1G22520 | -0.0587 | 0.0047 | 0.0000 | 0.7947 | 8.4858 | 0.0036 | 0.0361 |
| 262931_at |  | AT1G65700 | -0.0096 | 0.0309 | 0.2108 | 0.0065 | 8.7347 | 0.0031 | 0.0331 |
| 264686_at | ATSK41 | AT1G09840 | 0.0017 | -0.0351 | 0.8111 | 0.0010 | 8.2567 | 0.0041 | 0.0392 |
| 265301_s_at |  | AT2G13960 AT5G02320 | 0.1151 | 0.0043 | 0.0000 | 0.8641 | 10.9518 | 0.0009 | 0.0139 |
| 265385_at |  | AT2G20900 | 0.0392 | -0.0561 | 0.0001 | 0.0146 | 14.4111 | 0.0001 | 0.0032 |
| 265868_at | PUX2 | AT2G01650 | 0.0433 | -0.0301 | 0.0010 | 0.1457 | 8.9615 | 0.0028 | 0.0305 |
| 265934_at | EER5 | AT2G19560 | 0.0716 | -0.0224 | 0.0002 | 0.1299 | 15.2585 | 0.0001 | 0.0023 |
| 267044_at |  | AT2G34357 | -0.0540 | 0.0258 | 0.0046 | 0.0843 | 10.8636 | 0.0010 | 0.0144 |
| 267484_at | ATUBC2 | AT2G02760 | 0.0372 | -0.0055 | 0.0000 | 0.5794 | 14.2608 | 0.0002 | 0.0034 |
