# Supplementary material for: Meta‐analysis and meta‐regression of transcriptomic responses to water stress in Arabidopsis
Source: Plant J. 2016 Feb 12;85(4):548–60. doi: 10.1111/tpj.13124 (PMC4815425; doi:10.1111/tpj.13124)
Supplement: Supplementary file 11 — Table S9. Genes identified by the meta‐regression as having expression responses to water stress that are moderated by the type of experimental method, but which were not identified by even a single t test. [file TPJ-85-548-s011.docx]

**Table S9.** Genes identified by the meta-regression as having expression responses to water stress that are moderated by the type of experimental

method (deracination vs. mannitol vs. water withholding), but which were not identified by even a single t-test. The effects sizes ($\mu_{deracination},\mu_{mannitol},\mu_{water-witholding}$) calculated by the meta-regression for each method are provided.

| ID | Gene Symbol | AGI_TAIR | **$\mu_{deracination}$** | **$\mu_{mannitol}$** | **$\mu_{water-witholding}$** | QM | QMp | fdr (QMp) |
| --- | --- | --- | --- | --- | --- | --- | --- | --- |
| 244971_at |  |  | 0.0016 | -0.0308 | 0.0148 | 31.9926 | 0.0000 | 0.0000 |
| 245005_at |  |  | 0.0765 | -0.1058 | 0.0690 | 14.0062 | 0.0009 | 0.0148 |
| 246140_at |  | AT5G19910 | 0.0519 | -0.0350 | 0.0270 | 26.5348 | 0.0000 | 0.0001 |
| 246947_at | CYP71B11 | AT5G25120 | -0.2415 | 0.1373 | 0.0050 | 14.5135 | 0.0007 | 0.0121 |
| 247205_at | PROPEP2 | AT5G64890 | 0.0183 | 0.2277 | 0.0011 | 15.5669 | 0.0004 | 0.0080 |
| 247247_at |  | AT5G64650 | -0.0150 | 0.0046 | -0.0651 | 10.9623 | 0.0042 | 0.0477 |
| 247403_at |  | AT5G62740 | -0.0608 | -0.0224 | 0.0283 | 15.7182 | 0.0004 | 0.0076 |
| 247739_at |  | AT5G59240 | 0.0596 | -0.0549 | -0.0654 | 19.5146 | 0.0001 | 0.0015 |
| 247846_at |  | AT5G58100 | -0.0015 | -0.0359 | 0.0497 | 12.6557 | 0.0018 | 0.0246 |
| 248472_at |  | AT5G50860 | 0.1102 | 0.0009 | -0.0806 | 22.8661 | 0.0000 | 0.0003 |
| 248555_at |  | AT5G50340 | -0.1094 | -0.0530 | 0.0247 | 18.7740 | 0.0001 | 0.0020 |
| 248758_at |  | AT5G47620 | 0.0669 | -0.0462 | 0.0237 | 18.4947 | 0.0001 | 0.0023 |
| 248759_at |  | AT5G47610 | -0.0181 | -0.0296 | 0.0918 | 12.4539 | 0.0020 | 0.0267 |
| 248863_at | AGD9 | AT5G46750 | 0.0486 | 0.0153 | -0.0079 | 45.9238 | 0.0000 | 0.0000 |
| 249050_at |  | AT5G44290 | -0.0525 | 0.0284 | 0.0467 | 14.5379 | 0.0007 | 0.0121 |
| 249538_at |  | AT5G38840 | -0.0564 | -0.0488 | -0.0026 | 11.1965 | 0.0037 | 0.0435 |
| 249627_at | EMB1467 | AT5G37510 | 0.0483 | -0.0238 | -0.0003 | 38.0769 | 0.0000 | 0.0000 |
| 250188_at | COP13 | AT5G14250 | 0.0461 | -0.0250 | 0.0218 | 21.4944 | 0.0000 | 0.0006 |
| 250815_s_at |  | AT5G05040 AT5G05060 | 0.0166 | 0.0390 | -0.0022 | 20.0201 | 0.0000 | 0.0012 |
| 251090_at |  | AT5G01340 | 0.1754 | -0.0256 | 0.0296 | 11.0458 | 0.0040 | 0.0462 |
| 251136_at |  | AT5G01290 | 0.0195 | -0.0472 | 0.0155 | 16.9103 | 0.0002 | 0.0046 |
| 251186_at |  | AT3G62790 | 0.0018 | 0.0401 | -0.0366 | 13.1539 | 0.0014 | 0.0205 |
| 251210_at |  | AT3G62810 | 0.0330 | -0.0139 | -0.0293 | 20.4715 | 0.0000 | 0.0010 |
| 251581_at |  | AT3G58560 | -0.0068 | -0.0670 | 0.0146 | 14.2630 | 0.0008 | 0.0134 |
| 251843_x_at | ATHRGP1 | AT3G54590 | 0.0874 | -0.0047 | -0.0364 | 26.0000 | 0.0000 | 0.0001 |
| 251991_at | NF-YB10 | AT3G53340 | 0.0637 | -0.0008 | -0.0182 | 14.5843 | 0.0007 | 0.0120 |
| 252647_at |  | AT3G44620 | 0.0242 | -0.0149 | -0.0230 | 12.8432 | 0.0016 | 0.0229 |
| 252923_at |  | AT4G39050 | 0.0294 | -0.0774 | -0.0111 | 20.0213 | 0.0000 | 0.0012 |
| 252948_at | KAK | AT4G38600 | -0.0168 | -0.0434 | 0.0543 | 14.0968 | 0.0009 | 0.0143 |
| 253139_at | AKR2 | AT4G35450 | -0.0547 | -0.0429 | 0.0021 | 24.3435 | 0.0000 | 0.0002 |
| 253705_at | HXK1 | AT4G29130 | 0.0589 | -0.0476 | -0.0039 | 12.7585 | 0.0017 | 0.0236 |
| 254151_at |  | AT4G24390 | -0.1044 | 0.0429 | 0.0011 | 11.8435 | 0.0027 | 0.0336 |
| 254458_at | ATERDJ2B | AT4G21180 | 0.1509 | 0.0542 | -0.0962 | 21.0103 | 0.0000 | 0.0008 |
| 254913_at |  | AT4G11270 | -0.0667 | -0.0147 | 0.0358 | 20.3898 | 0.0000 | 0.0010 |
| 254937_at |  | AT4G10750 | 0.0239 | -0.0207 | 0.0211 | 20.4059 | 0.0000 | 0.0010 |
| 255985_at |  | AT1G34150 | 0.0693 | -0.0628 | 0.0071 | 16.3036 | 0.0003 | 0.0060 |
| 256932_at |  | AT3G22520 | -0.0865 | -0.0742 | 0.0321 | 25.9540 | 0.0000 | 0.0001 |
| 257285_at |  | AT3G29760 | -0.1604 | -0.0149 | 0.0200 | 14.8071 | 0.0006 | 0.0110 |
| 257666_at |  | AT3G20270 | 0.0744 | -0.0719 | -0.0400 | 16.6184 | 0.0002 | 0.0052 |
| 257857_s_at | RRP45a | AT3G12990 AT3G60500 | -0.0814 | -0.0252 | 0.0071 | 13.6699 | 0.0011 | 0.0166 |
| 258381_at | KINESIN-13A | AT3G16630 | -0.0254 | -0.0312 | 0.0295 | 27.0442 | 0.0000 | 0.0001 |
| 258848_at | DCL2 | AT3G03300 AT3G03305 | 0.0616 | 0.0199 | 0.0297 | 12.4105 | 0.0020 | 0.0272 |
| 259022_at | NS2 | AT3G07420 | 0.1208 | 0.0400 | -0.0473 | 14.1881 | 0.0008 | 0.0138 |
| 259950_at |  | AT1G71410 | -0.0584 | -0.0355 | 0.0208 | 19.7590 | 0.0001 | 0.0013 |
| 261426_at |  | AT1G18680 | 0.0404 | -0.0421 | 0.0111 | 20.1556 | 0.0000 | 0.0011 |
| 261689_at |  | AT1G50140 | -0.0362 | -0.0552 | 0.0257 | 18.6762 | 0.0001 | 0.0021 |
| 261770_at |  | AT1G76140 | -0.0056 | 0.0110 | -0.0317 | 14.5298 | 0.0007 | 0.0121 |
| 261800_at | PHV | AT1G30490 | 0.0337 | -0.0404 | 0.0373 | 13.0196 | 0.0015 | 0.0215 |
| 263391_at | MGDC | AT2G11810 | -0.2531 | -0.0270 | 0.2932 | 12.9054 | 0.0016 | 0.0223 |
| 263695_at |  | AT1G31220 | 0.0293 | 0.0467 | -0.0415 | 11.3962 | 0.0034 | 0.0403 |
| 264445_at |  | AT1G27290 | -0.0589 | 0.0148 | 0.0295 | 15.6307 | 0.0004 | 0.0078 |
| 264941_at |  | AT1G60680 | -0.2077 | -0.0382 | 0.0602 | 18.9591 | 0.0001 | 0.0019 |
| 266890_at | MTACP-1 | AT2G44620 | 0.0087 | 0.0109 | -0.0254 | 12.8230 | 0.0016 | 0.0230 |
| 267257_at |  | AT2G23080 | -0.0142 | -0.0059 | 0.0516 | 12.0010 | 0.0025 | 0.0318 |
| 267259_at |  | AT2G23090 AT2G44010 | -0.0066 | -0.0101 | 0.0321 | 12.5277 | 0.0019 | 0.0259 |
| 267506_at |  | AT2G45520 | -0.0363 | -0.0288 | 0.0016 | 12.7382 | 0.0017 | 0.0238 |
