# Supplementary material for: Meta‐analysis and meta‐regression of transcriptomic responses to water stress in Arabidopsis
Source: Plant J. 2016 Feb 12;85(4):548–60. doi: 10.1111/tpj.13124 (PMC4815425; doi:10.1111/tpj.13124)
Supplement: Supplementary file 13 — Table S11. Abscisic acid pathway‐related genes identified by the meta‐analysis. [file TPJ-85-548-s013.docx]

**Table S11.** ABA pathway related genes identified by the meta-analysis.

| **AGI** | **Name** | $\hat{\mu}$ | **FDR corrected p-value** |
| --- | --- | --- | --- |
| AT4G34000 | *ABF3* | 0.214 | 0.0026 |
| AT4G26080 | *ABI1* | 0.169 | 0.0001 |
| AT5G57050 | *ABI2* | 0.448 | <0.0001 |
| AT3G11410 | *AHG3* | 0.188 | <0.0001 |
| AT1G07430 | *AIP1* | 0.728 | <0.0001 |
| AT3G19290 | *AREB2* | 0.124 | 0.0040 |
| AT1G72770 | *HAB1* | 0.232 | 0.0005 |
| AT1G17550 | *HAB2* | 0.148 | 0.0004 |
| AT5G59220 | *HAI1* | 0.646 | <0.0001 |
| AT2G38310 | *RCAR10* | -0.318 | 0.0033 |
| AT5G46790 | *RCAR12* | -0.143 | 0.0041 |
| AT5G53160 | *RCAR3* | -0.100 | 0.0082 |
| AT4G33950 | *SnRK2-6* | 0.152 | 0.0033 |
| AT1G78290 | *SnRK2-8* | -0.164 | 0.0283 |
