# Supplementary material for: Meta‐analysis and meta‐regression of transcriptomic responses to water stress in Arabidopsis
Source: Plant J. 2016 Feb 12;85(4):548–60. doi: 10.1111/tpj.13124 (PMC4815425; doi:10.1111/tpj.13124)
Supplement: Supplementary file 14 [file TPJ-85-548-s014.docx]

## Supporting Information Legends

**Fig. S1.** Statistical properties of meta-analysis and meta-regression models. (a) The distribution of effect sizes ($\hat{\mu}$), P-values, and Q-values are provided for the grand means meta-analysis. (b) The distribution of effect sizes for genes in experiments testing different plant parts (tissues): shoots (leaves) and roots. The distribution of QMp values (significance of the effect of plant part) are also shown. (c) The distribution of effect sizes for genes from experiments where water deficit was caused by different treatments (deracination, water withholding, or mannitol). The distribution of QMp values (significance of the effect of treatment method) are also shown.

**Fig. S2.** Plot illustrating the effect of the experimental method on which genes respond to water stress, according to the meta-regression. Genes with a significant effect of experimental method (FDR corrected QMp < 0.05) were separated into three unique groups: genes that have their maximum expression change in response to deracination (first row; blue), genes that have their maximum expression change in response to mannitol (second row; green), and genes that have their maximum expression change in response to water withholding (third row; gold color). Within each row of plots, genes are sorted by its largest effect (max[$\mu_{deracination},\mu_{mannitol},\mu_{water-witholding}$], either up or down). Each gene is shown with a dot ($\mu_{deracination},\mu_{mannitol},\mu_{water-witholding}$) and horizontal line (95% CI).

**Table S1.** *Arabidopsis* genes with a significant differential expression response to water limitation, according to our meta-analysis of ten studies. Provided are the grand mean ($\hat{\mu}$) and its 95% confidence interval upper and lower bounds (ci.ub, ci.lb), the z-value (zval), p-value (pval), and a false-discovery rate corrected p-value (FDR pval).

[Table is provided as an excel file.]

**Table S2.** Genes with expression responses to stress that are significantly moderated by plant part (shoots versus roots), according to the meta-regression. (S2.1) Gene is upregulated in both shoots and roots, but less so in shoots than in roots. (S2.2) Gene is upregulated in both shoots and roots, but less so in roots than in shoots. (S2.3) Gene is upregulated in shoots and downregulated in roots. (S2.4) Gene is upregulated in roots and downregulated in shoots. (S2.5) Gene is downregulated in both shoots and roots, but more so in shoots than in roots. (S2.6) Gene is downregulated in both shoots and roots, but more so in roots than in shoots.

[Table is provided as an excel file.]

**Table S3.** Genes identified by the meta-regression with a significant effect of the type of water-stress treatment on the expression response. Provided is the Affymetrix gene ID, the grand mean expression response under deracination (Der.mean), mannitol (Man.mean), and water withholding (Ww.mean) treatments, the upper and lower bounds on those means (Der.ub Man.ub Ww.ub Der.lb Man.lb Ww.lb), the Q value of the model (QM), P-value (QMp), and a false-discovery rate corrected p-value (FDR QMp).

[Table is provided as an excel file.]

**Table S4.** Differentially expressed genes in each study (contrast), according to individual t-tests, with an FDR adjusted p value < 0.05. (S4A) GSE40061-root. (S4B) GSE40061-leaf. (S4C) GSE36789-root. (S4D) GSE36789-leaf. (S4E) GSE35258-seedling. (S4F) GSE15577-rosette. (S4G) GSE10670-leaf. (S4H) GSE6583-rosette.

[Table is provided as excel file.]

**Table S5.** Genes identified only by the meta-analysis and not discovered by the individual t-test contrasts, with $\hat{\mu}$ between -0.05 and 0.05.

[provided as an excel spreadsheet]

**Table S6.** Comparison of gene ontology frequencies of genes where the plant part (shoots vs. roots) moderates their expression, identified either by meta-regression or by comparison of t-test results across experiments. Class: BP=biological process, CC=cellular component. There were no significant differences between meta-analysis and t-test contrast frequencies in the molecular function category. An FDR corrected p-value based on Fisher’s exact test indicates the level of significant difference between the two frequencies. GO terms with higher frequencies in the meta-analysis are found at the top of the list, and GO terms with higher frequencies in t-tests are found at the bottom of the list.

**Table S7.** Comparison of gene ontology frequencies of genes where experimental method type (deracination vs. water withholding vs. mannitol) moderates their expression, identified either by meta-regression or by comparison of t-test results across experiments. Class: BP=biological process, CC=cellular component, MF=molecular function. An FDR corrected p-value based on Fisher’s exact test indicates the level of significant difference between the two frequencies. GO terms with higher frequencies in the meta-analysis are found at the top of the list, and GO terms with higher frequencies in t-tests are found at the bottom of the list.

[provided as an excel spreadsheet]

**Table S8.** Genes identified by the meta-regression as having a significant effect of organ covariate (roots vs. shoots), but which were not identified by even a single t-test. The effect sizes ($\mu_{roots},\mu_{shoots}$) and significance estimates calculated by the meta-regression for each organ are provided.

**Table S9.** Genes identified by the meta-regression as having a significant effect of method covariate (deracination vs. mannitol vs. water withholding), but which were not identified by even a single t-test. The effects sizes ($\mu_{deracination},\mu_{mannitol},\mu_{water-witholding}$) calculated by the meta-regression for each method are provided.

**Table S10.** Results from gene-ontology enrichment analysis for genes with a significant gene expression effect only in a single deracination study (GSE6583).

[provided as an excel spreadsheet]

**Table S11.** ABA pathway related genes identified by the meta-analysis.
